# Comparative Analysis of Vertebrate Diurnal/Circadian Transcriptomes

**DOI:** 10.1371/journal.pone.0169923

**Published:** 2017-01-11

**Authors:** Greg Boyle, Kerstin Richter, Henry D. Priest, David Traver, Todd C. Mockler, Jeffrey T. Chang, Steve A. Kay, Ghislain Breton

**Affiliations:** 1 Department of Integrative Biology and Pharmacology, McGovern Medical School, Houston, Texas, United States of America; 2 Division of Biological Sciences, University of California San Diego, La Jolla, California, United States of America; 3 Donald Danforth Plant Science Center, St. Louis, Missouri, United States of America; McGill University, CANADA

## Abstract

From photosynthetic bacteria to mammals, the circadian clock evolved to track diurnal rhythms and enable organisms to anticipate daily recurring changes such as temperature and light. It orchestrates a broad spectrum of physiology such as the sleep/wake and eating/fasting cycles. While we have made tremendous advances in our understanding of the molecular details of the circadian clock mechanism and how it is synchronized with the environment, we still have rudimentary knowledge regarding its connection to help regulate diurnal physiology. One potential reason is the sheer size of the output network. Diurnal/circadian transcriptomic studies are reporting that around 10% of the expressed genome is rhythmically controlled. Zebrafish is an important model system for the study of the core circadian mechanism in vertebrate. As Zebrafish share more than 70% of its genes with human, it could also be an additional model in addition to rodent for exploring the diurnal/circadian output with potential for translational relevance. Here we performed comparative diurnal/circadian transcriptome analysis with established mouse liver and other tissue datasets. First, by combining liver tissue sampling in a 48h time series, transcription profiling using oligonucleotide arrays and bioinformatics analysis, we profiled rhythmic transcripts and identified 2609 rhythmic genes. The comparative analysis revealed interesting features of the output network regarding number of rhythmic genes, proportion of tissue specific genes and the extent of transcription factor family expression. Undoubtedly, the Zebrafish model system will help identify new vertebrate outputs and their regulators and provides leads for further characterization of the diurnal cis-regulatory network.

## Introduction

Life evolved under a recurring alternation of light and darkness currently lasting about 24 hours [1]. The circadian clock, a time-tracking anticipatory system, evolved during the last billion years into a transcriptionally based mechanism consisting in multiple feedback loops [2,3]. Depending on the organism, different inputs such as light or feeding can entrain the oscillator and regulate physiological outputs such as meal time, sleep phase, flower orientation to attract pollinator and so on [4,5]. Thus the oscillator’s evolution must have involved changes in core clock gene bodies and concomitantly in a wide array transcription factor (TF) binding sites (also referred to as cis-regulatory motifs) associated with output regulation.

The recent advent of genomic techniques was instrumental in determining the large size of the rhythmic output network comprising 6% to 20% of the expressed genome depending on the cell type or tissue and environmental conditions [6–8]. Irrespective of the rhythmic transcript scoring approach or sampling rate, most of the studies identified that around 1–2 thousand genes are rhythmically transcribed, and all the expression phases are covered with larger cohorts of transcripts around dawn and dusk [9–11]. Larger profiling projects covering 12 different tissues or 11 different environmental conditions revealed that 43% and 89% of the genes cycle in at least one tissue or environmental conditions respectively [9,12]. Thus the current understanding is that depending on the environmental condition or tissue type, almost all genes can be rhythmically expressed.

Large scale rhythmic gene expression data has been used to identify known and novel cis-regulatory motifs potentially involved in the regulation of the larger output network [9,13,14]. Chromatin-immunoprecipitation techniques paired with next generation sequencing were also instrumental in confirming that core clock transcription factors such as BMAL1, CLOCK, REV-ERB are rhythmically bound at thousands of sites in the genome potentially participating not only in the entrainment of the oscillator but in the regulation of its output as well [11,15]. Although the core clock mechanism has been modeled using a multitude of approaches [16], a detailed level of the connectivity map of the output network will require a much larger effort based on its sheer size and multiple variations.

Zebrafish is an important model for the characterization of the core circadian clock and its inputs [17,18]. Efforts are also underway to determine its output network [19–21]. Our long term goal is to build a vertebrate consensus circadian output network using the wealth of data available from mammals [22,23]. With the aim of using Zebrafish to test hypothesis *in vivo* for a better understanding of diurnal physiology, we wanted to investigate the similarities between the diurnal/circadian genes in Zebrafish and mouse. To leverage the multitude of circadian mouse liver datasets generated by the community [22,23] we aimed to profile the same organ in the vertebrate Zebrafish *Danio rerio*. Zebrafish is a diurnal animal with a well-annotated and complete genome making it an ideal model for comparative transcriptomics [24,25]. At half the size of the human and mouse genomes (1.5Gb as compared to 3.4Gb), the Zebrafish contains a similar number of genes (25K as compared to 20 and 22K respectively). Our study reveals that the liver rhythmic transcriptome is of similar size as its mammalian counterparts, with phases covering the whole 24hr spectrum. Comparative analyses between other Zebrafish time courses revealed that the core clock genes are expressed at the same time of the day and that large groups of genes phase shift between the adult liver and the larval stage. Comparison with mouse and human time courses also highlighted similarities between the core clock set and shifting groups of output genes. Taking advantage of a large 12 mouse tissue rhythmic transcriptome dataset, we investigated potential features associated to the liver-like similarities of the Zebrafish liver dataset [12]. The comparative approach identified global differences and similarities with the mouse liver and other datasets. Phase differences were found in clusters co-expressed in different datasets suggesting that the output network is not necessarily sequential waves of gene clusters but could rather be discontinuous with some clusters expressed with delay or advance to others depending on the conditions.

## Materials and Methods

### Ethics statement

This study was carried out in strict accordance with the recommendations in the Guide for the Care and Use of Laboratory Animals of the National Institutes of Health. The protocols for animal studies were approved by the Animal Welfare Committee (IACUC) of the McGovern Medical School at the University of Texas Health Science Center Houston (permit numbers: AWC-10-158 and AWC-13-124) and the University of California San Diego Institutional Animal Care and Use Committee (permit number: S04168). All efforts were made to minimize suffering or distress. Euthanasia methods were immersion in lethal dose of buffered tricaine solution.

### Zebrafish stocks

Zebrafish AB line were mated, staged and raised as described [26]. For the expression profiling using array, a week prior to the time course, 3–6 months old adult Zebrafish males were separated into group of 4 for sampling. In order to eliminate possibilities of brine shrimp RNA sampling from the liver tissue, the fish had their last meal at noon a day prior to the experiment (skipping the last 5PM meal). The next morning, starting at light ON (LD24 7:00AM– 14hrs light/10hrs dark), fish were euthanized in tricaine (0.15%) and livers from four males were extracted using scalpel, surgery scissors and dissecting microscope and pooled in a single tube. Livers were sampled at 4hr intervals for 2 continuous days. The two dissections in darkness (e.g. LD40 and LD44 for day 1 and LD64 and LD68 for day 2) were performed with dim red light attached to the dissecting microscope.

For expression profiling using the QuantiGene platform (Affymetrix), larva were raised in groups of 40 embryos in petri dish containing standard embryonic buffer under light/dark regime of 12:12 and temperature of 28.5°C in a Percival I-41LL incubator. Day 5 and day 6 larva were concentrated using a 50mL conical tube with a mesh bottom. The tubes were placed directly in E3/tricaine solution for euthanasia. Day 5 and Day 6 larva were sampled in biological duplicates at 2hr intervals on the same day using infrared goggles to handle dish and tube during the night period. Following euthanasia, larva were gently transferred with a 3mL transfer pipette into a microtube and frozen in liquid nitrogen (this procedure was performed under light).

### RNA extraction

For expression profiling using microarray, liver samples were immediately submerged in 1mL of RNALater solution (Qiagen). All samples were processed together at the end of the time course for RNA extraction. Tissues were homogenized using a Retsch MM200 mixer mill and glass beads. Total RNA was extracted using RNA extraction kit from Qiagen using supplier's recommendations for liver samples.

### Microarray design

The 12-plex arrays were custom-designed by NimbleGen (OID 22743). The 60-mer probes were designed in June 2009 based on transcript sequences annotations from *Danio rerio* version Zv8. Four probes were designed for 27,082 out of the 28,717 annotated transcripts in Zv8.

### cDNA synthesis and hybridization for microarray

RNA quality was tested with the Agilent 2100 Bioanalyzer. cDNA synthesis and labeling, as well as array hybridization, scanning and RMA data processing were performed by the UCSD/VA GeneChip core. Transcript expression data were a combination of all four 60-mer probes in each probeset.

### Transcript profiling using Affymetrix QuantiGene

Tubes containing 40–45 frozen Zebrafish larva were supplemented with 300uL of QuantiGene homogenizing solution following the protocol for direct preparation of homogenate from animal tissue (Affymetrix). The solution was homogenized using three 3.5mm glass bead and disrupted using a BioSpec Mini-Beadbeater 8 with 3 pulses of 5 seconds. The sample was then immediately processed without RNA extraction or cDNA synthesis using the QuantiGene protocol for Cell lysates. Beads were read using a Luminex 200 station at the Brown Foundation Institute for Molecular Medicine Flow Cytometry Service Laboratory Core. Biological duplicates were collected and each sample was processed in technical duplicates. The raw intensity readings from the QuantiGene Plex assay technical duplicates were first averaged, background-subtracted and then normalized to the *timeless* reference gene. We found that the timeless gene displayed minimal variability over the 24hr LD cycle and at a mid-range expression level well suited for a reference gene. For plotting graphs, biological samples were averaged and standard deviation was calculated using MS Excel and plotted using Prism. T-test was used to calculate statistical significance (p value) between troughs and acrophases.

### Microarray analysis

The data was initially analyzed using HAYSTACK [27]. HAYSTACK is a model-based pattern-matching algorithm, which compares a collection of diurnal/circadian models against microarray time-course data to identify cycling genes. It has been implemented in perl, and uses least-square linear regression for each gene against all model cycling patterns with 24 possible phases. Permutations were performed and the top 5% hits were selected with a correlation cut-off of 0.81 [27]. HAYSTACK can be accessed online at (http://haystack.mocklerlab.org).

To annotate the microarray probes with the most recent assembly of the Zebrafish genome (Zv10), we aligned the probe sequences against the Zv10 genome using blat [28,29]. Only probes assigned to a single genomic locus were kept. From those, only complete probesets of 4 probes from the original design were kept for further analysis. We then annotated the probes with Ensembl IDs based on their genomic loci [30]. We found that numerous probes were assigned to a unique locus but had multiple gene annotations in Zv10. In order to maximize our chances of identifying potential mammalian orthologs, we created non-redundant entries merging the probeset ID with a unique gene ID into a probeset-gene ID. Expression data were duplicated to reflect the expanded probeset-gene ID list. Only probeset-gene entries assigned to the 4 loci from the original design were maintained.

Previous results from HAYSTACK were trimmed of older probesets containing inaccurate probe assignments to reflect Zv10. To complement the HAYSTACK results, we also performed a rhythmic gene search using JTK_CYCLE [31] (p < 0.05, 20 ≤ period (τ) ≥ 28hr). The R version of JTK_CYCLE was downloaded from the Hughes lab website (http://openwetware.org/wiki/HughesLab). The data used to run JTK_CYCLE was the Zv10-trimmed dataset making the results compatible with the HAYSTACK data.

### Data mining

All phase datasets were analyzed using MS Excel and Filemaker Pro. In order to perform comparative analysis between *D*. *rerio* and *M*. *musculus*, the zebrafish transcript data was first transformed to their respective gene entry. As several rhythmic transcripts were found to be associated to the same genes, we calculated the average phase taking into account special cases of dawn-phased (around ZT23 and ZT0) genes and provided manually curated corrections depending on the timing of the average (e.g. The average of ZT2 and ZT22 is not ZT12 but ZT0 (manual correction is ZT2 = ZT26)). Venn diagram and Tanimoto-Jaccard indexes were generated using Venny (http://bioinfogp.cnb.csic.es/tools/venny/) and proportional graphic were generated using VennDIS [32].

## Results and Discussion

Transcriptomic studies were instrumental in revealing how extensive the circadian clock control is over genome-wide gene expression [6,7,10]. Studies using oligonucleotide array platforms and next generation sequencing techniques consistently show that ~10% of the transcriptome in a specific cell type or tissue displays diurnal or circadian rhythms lasting approximately 24hrs [8,10,11]. Lower harmonic rhythms were also uncovered [10]. Numerous computational strategies were devised in order to identify rhythmic genes from long time courses datasets [33]. Benchmark comparisons have compared the most common [34].

Due to its numerous metabolic functions, the mouse liver is the most diurnally profiled organ [35]. Comparative studies between mouse datasets helped identify commonly expressed genes and their potential role in the circadian system [14]. The large number of rhythmic genes identified in each dataset suggests that the circadian transcriptome is a large cis-regulatory network with the core clock regulating a multitude of outputs. As its characterization moves forward, the vertebrate output network may be similar to the Arabidopsis network with simple output without any feedback inhibition on the core oscillator [36] to more complex ones with downstream feedback [37,38]. To this day, only a small fraction of the outputs have been characterized. One recent observation from the comparison of mouse liver datasets is the relatively low level of overlap between datasets [39,40]. This result could be due to technical differences in sampling rates, the accuracy of the different scoring algorithms, or transcriptomic platforms. Although all these parameters certainly have an effect from one dataset to the other, it was also proposed that the output network is pervasive and that unaccounted biological parameters may be at the source of part of these transcriptome differences [3,40]. If this hypothesis is correct, it would suggests that the output network can produce several different outcomes depending on different conditions. This was shown to be the case using large environmental changes of light and temperature in plants and feeding in mouse liver [9,41]. The question would not simply be how many genes are oscillating but how many genes can oscillate in a specific condition. Thus, there is potentially an array of outputs for each tissue depending on the conditions.

**Fig 1:**
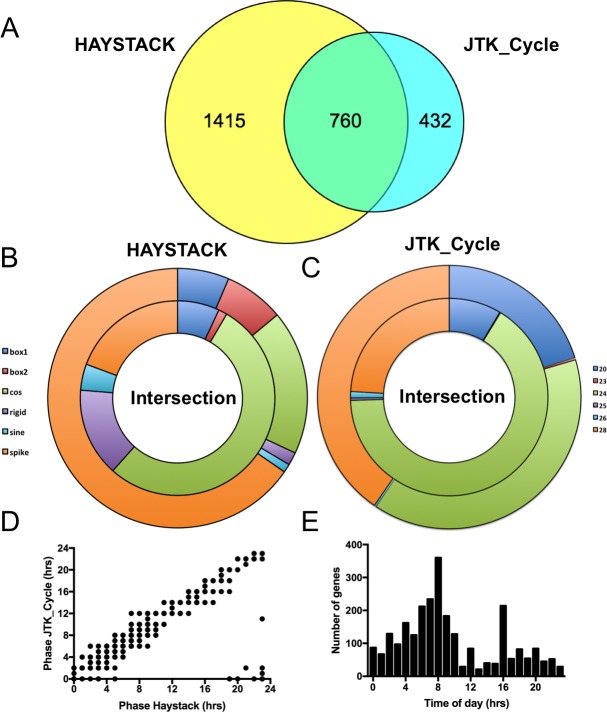
Rhythmic Zebrafish liver transcripts. (A) Venn diagram of rhythmic Zebrafish liver transcripts identified using HAYSTACK and JTK_Cycle. (B) Difference in the shape of expression profile between transcripts identified by HAYSTACK alone or by both HAYSTACK and JTK_Cycle (Intersection in Fig 1A). Shapes listed in legend are described in [9]. (C) Difference in period between transcripts identified by JTK_Cycle alone or by both HAYSTACK and JTK_Cycle (Intersection in Fig 1A). Numbers in legend correspond to period in hours. (D) Scatter plot of phase of genes identified by HAYSTACK and JTK_Cycle. (E) Rhythmic transcript count by phase of the day.

For the present study, we wanted to expand the comparative analysis performed by others using mouse and rat liver datasets and expand it to a lower vertebrate species [14]. Establishing parallels beyond the core oscillator between Zebrafish and rodent output networks could open up avenues for further exploration of the network using unique features of the Zebrafish model. We selected Zebrafish *Danio rerio* and performed a two days time course. At an interval of 4 hours, we dissected and pooled 3–4 adult male fish livers under light/dark (14hrs light/10hrs dark starting at light ON (7AM)). We use a custom Nimblegen 12-plex array designed from the Zv8 *D*. *rerio* genome version. The array annotations were subsequently updated by realigning each probe to the current Zv10 genome version [24]. The array interrogates 23,635 transcripts using probesets of four independent probes. In total, the array interrogates 19,683 unique Zv10 Zebrafish genes with at least one probeset (GEO GSE87659). Two different algorithms were used to identify cycling genes. Out of the 23635 probesets, 2175 were determined to be rhythmic by HAYSTACK (9.2% of total probesets) and 1192 (5% of total) by JTK_Cycle [27,31]. Globally, 760 transcripts were common for a total of 2607 rhythmic transcripts (11% of total)(Fig 1A and S1 Table).

As in our previous studies, the use of HAYSTACK was selected in order to identify rhythmic transcripts with various types of shapes [9,27,42]. In addition, we also used JTK_Cycle, one of the most common algorithms [10,31]. Analysis of HAYSTACK specific transcripts compared to the transcripts identified by both algorithms (intersection) revealed that HAYSTACK identified a larger proportion of spike shape expression patterns (Fig 1B). Box-like shapes were also overrepresented (Fig 1B). When performing a similar analysis with results from JTK_Cycle, we found that this algorithm identified a higher proportion of transcripts with period of 20 and 28 hours than HAYSTACK (Fig 1C). Thus both algorithms using standard settings identified rhythmic transcripts with their own specific features. Analysis of the 760 common transcript phases revealed that both algorithms performed similarly (Fig 1D) and that 99% of the phases were found within the 4hrs window of the sampling frequency (S1B Fig) (All phases is R^2^ of 0.94, calculated from circular adjusted phases (S1A Fig)). Based on these results, datasets were merged to generate a broad shape and period dataset of 2607 cycling transcripts. Phase analysis of the ensemble revealed that they are covering all phase of the day (Fig 1E) with a maximum number of transcripts peaking prior to dusk (ZT8). Although a similar number of rhythmic transcripts were identified in a Zebrafish larval time course (2856) [43], the time of day at which the majority of transcripts were expressed were different with peaks at dawn (CT0) and post-dusk (CT16) [43]. This data represents a first difference between the larval and adult Zebrafish datasets.

**Fig 2:**
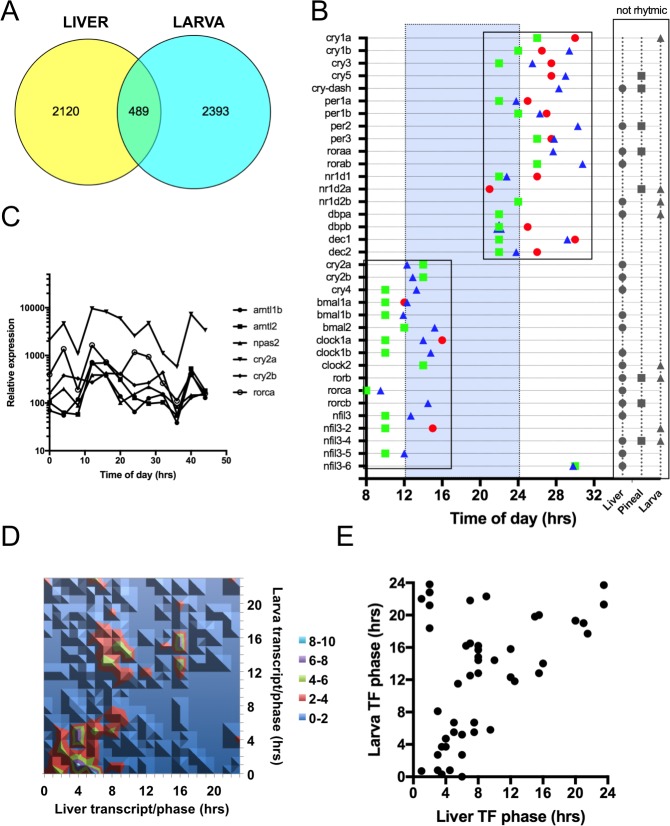
Comparison between Zebrafish larval and liver time courses. (A) Venn diagram of the rhythmic transcripts identified in Zebrafish larva and liver. The larval genes are the common rhythmic genes from 5d old larva collected every 4hrs under LD (14:10) and DD (2882 ENSEMBL genes)[43]. (B) Expression phases of clock genes in zebrafish pineal gland, larva and liver. Red circle: liver; Green square: pineal gland (from [44]; Blue triangle: larva. Grey symbols correspond to arrhythmic genes. (C) Diurnal profiles of selected arrhythmic liver clock genes from panel B. (D) Contour plot of the 489 common cycling genes between the liver and larval time courses. Color code in legend represents the number of genes at each phase intersection. (E) Scatter plot of the phases of all cycling TF genes between the liver and larval time courses.

As a first step in our comparative transcriptomic study, we compared our dataset with that of Li et al [43] from 5d old Zebrafish larva. In this particular case the authors performed a pair of time courses, one under light/dark cycle and one in constant darkness. The authors combined the results from both time courses and identified 2856 common diurnal/circadian rhythmic transcripts [43]. In order to provide a common denominator for the purpose of comparative analysis, we converted all transcript annotations into common unique ENSEMBL gene annotations (S1 Table). Thus after conversion, the 2607 transcripts could be assigned to 2609 genes using the Zv10 annotation from ENSEMBL (Some transcript were associated to the same genes and some were associated to multiple ones). The combined larva time courses contained 2882 Zv10 ENSEMBLE genes. Out of those, 489 genes were common with the Zebrafish liver dataset (Fig 2A). As an estimate of the extent of the overlap, we calculated the Venn diagram Tanimoto-Jaccard index ((Intersection/Union)*100). This index takes into account the size of both ensembles and of the overlap. A theoretically perfect overlap would give a score of 100. As examples, the index calculated below between liver time courses from the same research group was 29.7 [10,12] and the one between a liver and a hypothalamus datasets was 1.4 [12]. The index between the Zebrafish liver and larval time courses is 9.8. This similarity score from time courses performed using different tissues from two different labs using two different array platforms could be considered moderately similar.

Plotting the phase of expression for all core clock genes from both datasets revealed a common timing regulation organized in two clusters (Fig 2B). The addition of a third dataset from the Zebrafish pineal gland shows the same pattern (Fig 2B) [44]. A cluster of genes was expressed at dawn and another at dusk. The average phase of the dawn and dusk clusters is ZT1.6 and ZT12.7 respectively. The dawn group contains *cry1*, *3*, *5* and *cry-dash*, as well as *per1*, *2*, and *3*, *rora*, *reverb*, *dbp* and *dec1* and *2*. One or two genes from each group were found to cycle at dawn in each time courses. From a total of 18 listed genes, 13 are cycling in the pineal gland, 14 in the larva and 12 in the liver. Dawn phases were also reported by other groups for these genes [45–47]. The dusk group contains *cry2* and *4*, *bmal1* and *2*, *clock*, *rorb* and *c* and *nfil3* (*e4bp4*) (Fig 2B). In this case, the pineal and larva time courses contain a gene of each group but the liver time course does not. Neither *cry2* or *4*, nor *rorb* or *c* were found to cycle in the liver. Several other groups reported similar phases for gene of the dusk cluster [48–49] From 17 clock genes listed, 13 are cycling in the pineal gland, 12 at the larval stage and only 3 in the adult liver. Close inspection of the liver transcript profiles revealed double peak expression pattern that would not have been identified by the two algorithms used (Fig 2C).

Since rhythmic core clock genes are expressed in two major clusters with similar phases, we plotted the phase of the 489 common cycling genes to determine if there was a similar trend (Fig 2D). The comparison of both datasets revealed that only 58% of the genes have similar phases within the boundaries of the 4hrs sampling frequency)(The calculated phase similarities had a R^2^ of 0.56 (S1C and S1D Fig)). The majority of the genes (347) fell into 3 clusters (Fig 2D). In the larva time course, the largest cohorts of genes are cycling with a peak during the early day (ZT0-ZT7) or the early night (ZT10-ZT18)(Fig 2D). The early day group encompasses a similar phase spectrum in the liver time course (ZT0-ZT10). However, the larval dusk group (145 genes) is split in two groups in the liver time course being expressed at the end of the day (98 genes-ZT5-ZT10) or at the beginning of the night (47 genes-ZT14-ZT18)(Fig 2D). This result suggests differential regulation of at least one large group of genes between the liver and the larva under light/dark conditions. Since the core clock genes have similar phases between the larval and liver time courses, it is possible that this asynchronous group is regulated by different sets of rhythmic TFs. To determine if some regulators were being regulated with a similar phase difference, we extracted and plotted the TFs from the ensemble of 489 common rhythmic genes. Using the TF domains defined by Armand et al [50], we generated a list of all transcription regulators in Zebrafish (S2 Table). Among those, several had phase changes similar to the three clusters suggesting potential regulators (Fig 2E).

After comparing the Zebrafish liver time course with a time course from the same species, we went ahead and performed the intended comparative analysis at the origin of this study by comparing liver time courses from Zebrafish and mouse. From a circadian clock perspective, the mouse liver is the most characterized organ at the transcriptional level [8,10]. In order to pursue our comparative transcriptomics study against the available mouse liver time courses, we converted all Zebrafish rhythmic genes into their mouse putative orthologs (S1 Table). Among all 2609 rhythmic genes, 449 did not have an associated mouse ortholog in BioMart. From the remaining 2160 genes, several had more than one potential mouse orthologs. By compiling a list containing all non-redundant mouse orthologous genes, we identified 2530 unique orthologs (S1 Table).

**Fig 3:**
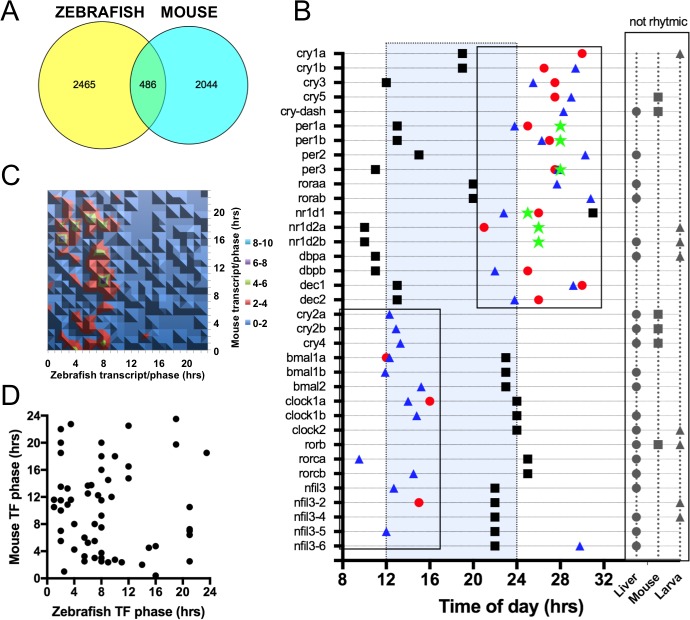
Comparison of Zebrafish and mouse liver rhythmic genes. (A) Venn diagram of the rhythmic transcripts identified in Zebrafish and mouse livers. The Zebrafish liver data were converted into putative mouse ENSEMBL orthologs (2951 unique genes) and compared with the Hughes et al. dataset [10](1hr resolution for 2d in DD from CircaDB)(2530 Unique ENSEMBL genes). (B) Expression phase for clock genes in zebrafish, mouse and human. Red circle: Zebrafish liver; Blue triangle: Zebrafish larva; Black square: Mouse liver; Green star: Human hair follicle cells [51]. Grey symbols correspond to arrhythmic genes. (C). Contour plot of the 486 common cycling genes between the Zebrafish and mouse liver time courses. Color code in legend represents the number of genes at each phase intersection. (D) Scatter plot of the phase of all cycling TFs genes between the mouse and Zebrafish time courses.

As a starting point for comparative transcriptomics, we used the Hughes et al. [10] dataset consisting of the highest sampling rate liver time course to date (each hour for 48 hours) (Fig 3A). The phase information for the mouse dataset was obtained from the CIRCA database using the same search parameters used in Hughes et al [10,22]. We identified 4744 cycling probesets. From this list, a BioMart analysis identified 4437 Ensembl gene ID. By restricting the analysis to the period range covered in our own study (20 and 29 hours), we identified 3878 cycling mouse genes. Since several probesets were assigned to the same gene, the phase score were averaged by paying close attention to phases close to dawn (CT23 and CT0). The final list contained 2951 non-redundant mouse cycling genes. Using the two lists of Ensembl ID genes from Zebrafish and mouse livers, we identified 486 common genes (Fig 3A)(Tanimoto-Jaccard index *100 = 9.7). As compared with the analysis of the Zebrafish liver and embryo time courses, the groups are of similar size and the intersection size is also of a similar magnitude. The fact that the Tanimoto-Jaccard index is almost identical between studies is suggesting that the Zebrafish liver time course is as similar to the larval dataset as it is to the mouse liver one.

As a step further in mapping the similarities between the zebrafish and mouse diurnal liver transcriptome, we identified all cycling clock genes and compared their phase with our previous analysis (Fig 3B). The mouse core clock gene phases are almost all anti-phasic to their zebrafish counter parts. Human core clock genes identified from hair follicle cells follow the same trend as the Zebrafish data (Fig 3B) [51]. This difference may be due the diurnal/nocturnal activity pattern between these animals [52,53] although clock genes were shown to not always follow this pattern between nocturnal and diurnal species of fish [54,55]. The Zebrafish dawn cluster that had a phase of ZT1.6 in Fig 2B has now a phase of ZT13.6 in mouse while the dusk cluster phased at ZT12.7 is now expressed at ZT21.8 (Fig 3B). The only two exceptions that are not following this anti-phasic trend are *cry1* and *rora* that are expressed at the early dawn phase of CT19 and CT20 respectively (Fig 3B). If we exclude these two genes from the dusk cluster (now ZT11.3), two features are similar between the two networks. The phase difference between their respective dawn and dusk core clusters is both at the opposite side of the 24hrs continuum. The timing between the two clusters in Zebrafish is 11.1hrs during the day and 12.9hrs during night while it is 13.5hrs during the day and 10.5hrs during the night in mouse.

An analysis of the common cycling genes between the Zebrafish and Mouse liver time courses revealed a very different picture than the core clock gene subset (Fig 3C). Similarly to what was observed for the liver/larval analysis, the fish/rodent liver comparison also revealed that not all genes have the same phase. Only 36% of the genes have similar phases within the boundaries of the 4hrs sampling frequency (Fig 3C)(R^2^ for all phases was equal to 0.40, S1E and S1F Fig). While the comparison between Zebrafish larva and liver showed overlap with day and night genes between time courses, in the case of the mouse and zebrafish livers, the overlap identifies clusters only during the day. Three clusters can be identified (Fig 3C). The first one encompasses ZT6 and ZT10 in Zebrafish and ZT0 to ZT9 in mouse and contains 106 genes. The second one includes ZT0 to ZT10 in Zebrafish and ZT10 to ZT16 in mouse and contains 146 genes. The third cluster covers ZT2 to ZT10 in Zebrafish and ZT17 to ZT23 in mouse. Based on the anti-phasic nature of the core clock gene clusters in Fig 3B, the second and third clusters are potentially regulated by clock genes. Cluster 1 contains genes expressed in the morning under both conditions (Fig 3C). Plotting the phase for all the TFs identified from this dataset identify 11 potential regulators in the ZT6-10/ZT0-9 time frame (Fig 3D).

**Fig 4:**
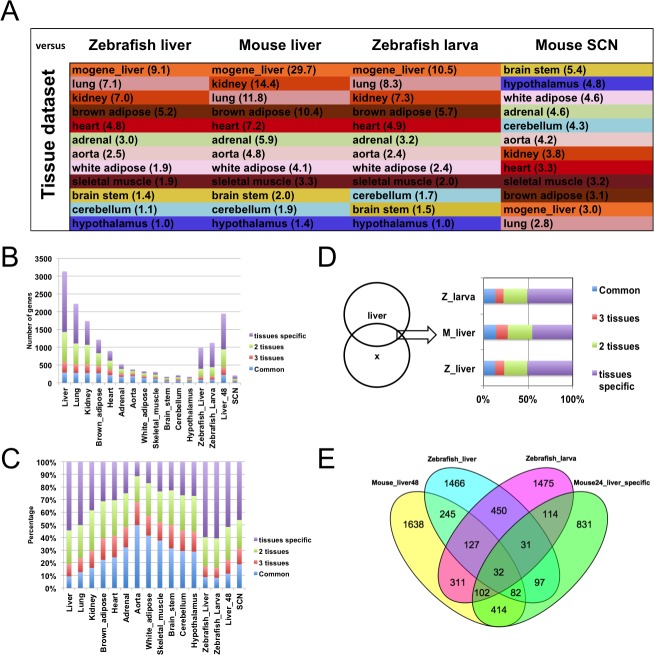
Zebrafish and mouse liver rhythmic genes compared to 12 mouse tissue time courses. (A) Color-coded table displaying the ranking from highest to lowest of the Tanimoto-Jaccard index from the comparison of the Zebrafish or Mouse liver time courses against 12 different tissues time courses from Zhang et al. [12]. As controls, Zebrafish larval and Mouse SCN datasets were also compared with the 12 tissues ensemble. (B) Number of common (present in 4 to 12 tissues) or tissue specific genes (present in 1 to 3 tissues) in the 12 tissues and surveyed datasets. (C) Proportion of common (present in 4 to 12 tissues) or tissue specific genes (present in 1 to 3 tissues) in the 12 tissues and surveyed datasets. (D) Proportion of common (present in 4 to 12 tissues) or tissue specific genes (present in 1 to 3 tissues) in the intersection of the Venn diagram between the mouse liver and the Zebrafish larval, liver or Mouse liver (Liver_48) time courses. (E) Venn diagram between the complete mouse liver (Liver_48), Zebrafish liver and larval datasets and the liver specific subset from the mouse liver in [12].

To continue our analysis of these datasets, we took advantage of a second study performed by the Hogenesch group [12] comparing cycling genes in 12 mouse organs. This rich dataset was used to determine if the Zebrafish liver time course is closer to the mouse liver time course than any other mouse tissue? Using the CircaDB database, we generated the list of cycling genes in every tissues analyzed in Zhang et al. [12] (including an additional liver time course identified as mogene_liver in CircaDB). We compared the Zebrafish liver time course to each tissue datasets and generated Venn graphs for each comparison. For each graph, the Tanimoto-Jaccard index was calculated and the score ranked from the highest to the lowest (Fig 4A). We performed the same analysis using the mouse liver 48 samples dataset and generated a similar ranking [10]. The tissue time course most similar to the Zebrafish and Mouse liver time courses was the mogene_liver time course. The ranking from both analyses were also very similar (Fig 4A). Despite a moderately low overlap between the Zebrafish and mouse liver time courses (Fig 3A), the Zebrafish dataset contains characteristics making it closer to mouse liver than any other tissue (Fig 4A). As additional controls, we also tested the similarities with Zebrafish larval stage and Mouse suprachiasmatic nucleus (SCN from CircaDB) time courses [22,43]. The larva dataset was more similar to the liver dataset than any other tissue. In addition, its ranking was also very similar to the mouse and Zebrafish livers rankings. This result may be associated to the high metabolic activity in developing larva and also possibly in other tissues such as the kidney and lung. In the case of the SCN time course, the top similarity was the brainstem dataset and the ranking was almost the reverse of the other three comparisons showing that not all comparison has liver as its top score.

The Tanimoto-Jaccard index for the Zebrafish liver and larval datasets against the liver time course were 9.1 and 10.5 respectively (Fig 4A). In order to investigate which features provide the liver identity to the Zebrafish liver time course and determine if these are different than the features associated to the larval time course, we again took advantage of the 12 tissues datasets from Zhang et al [12]. This large dataset contains a total of 7225 unique cycling genes. We organized these genes in 12 bins ranging from being present only in a single tissue to present in all tissues. Searching genes common between circadian datasets is often used as a strategy to identify novel core clock genes since their expression is expected to be maintained in each circadian clock-regulated datasets [14]. We organized tissue genes in 4 larger groups. The first group contains the most common genes (404 genes) present in 4 to 12 tissues. The remaining three groups contain tissue specific genes present in 1, 2 or 3 tissues (4581, 1553, and 499 genes respectively). Using theses new groups, we analyzed the 12 tissues and displayed the number and proportion for each one (Fig 4B and 4C). The ranking obtained from the Zebrafish liver analysis was used to organize the tissue in Fig 4B and 4C. As observed by Zhang et al. [12], the liver time course contains the highest number of rhythmic genes (Fig 4B) and 9% of these are common rhythmic genes (289 out of the 404 common genes from the whole ensemble) (Fig 4C). Fig 4B and 4C, reveal a trend between tissues. Tissues with large amount of cycling genes contain a large proportion of tissue specific genes (organ tissues) and tissues with low amount of cycling genes (brain areas) contain a higher proportion of common rhythmic genes. The Zebrafish liver and larval time courses share a high level of similarities with the organ tissue by their number of cycling genes and proportion of common and tissue specific genes (Fig 4B and 4C). The mouse liver time course from Hughes et al. [10] shares the same features as the Zebrafish time courses while the SCN time course only share a low amount of cycling genes with its brain area-enriched time course since the proportion graph is similar to organ tissue datasets (Fig 4B and 4C).

Although the previous analysis helped differentiate two groups of tissue, it did not help determine possible differentiating features between the Zebrafish liver and larval time courses. If the overall ensembles are similar, does the subset of genes common with the mouse liver time course have differentiating features? We performed a similar tissue specific gene analysis on the gene found at the intersection of each Venn diagrams (Fig 4D). Our analysis revealed that the intersection between the liver time course (mogene_liver) and both time courses have also similar common/tissue specific genes profiles (Fig 4D). Does the Zebrafish liver time course contain more liver specific genes than the Zebrafish larval time course? The 12 tissues datasets can also be utilized to identify genes that are specifically expressed in the liver. Out of 7225 genes, 1703 are expressed specifically in the liver. From those, 630 are also expressed in the 1hr sampling rate mouse liver time course from Hughes et al. [10]. From these 630 liver specific genes, 32 are common between the liver and larval time courses, 82 genes are only present in the liver time course while 102 are only present in the larval time course (Fig 4E). Based on this analysis, the Zebrafish liver time course contains less liver specific genes than the larval time course. This result suggests that the similarity shared by these three datasets is not necessarily due to their liver-like identity.

**Fig 5:**
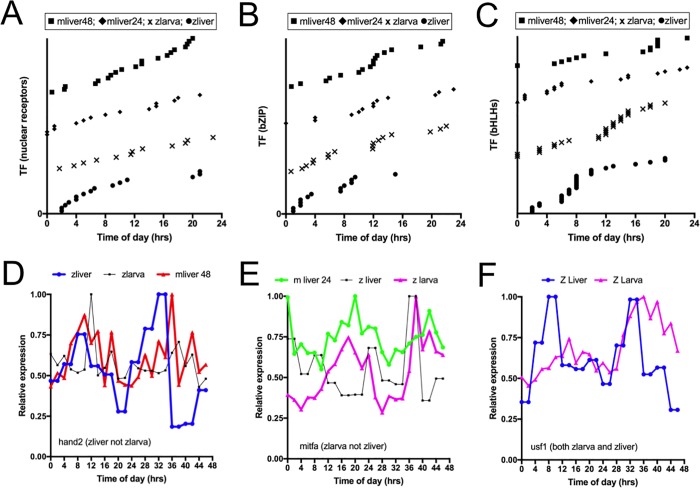
E box, D box and RRE regulator phases in mouse liver and Zebrafish tissues. (A) Phase of rhythmic nuclear receptor transcripts expressed in the mouse liver. 1hr sampling rate time course: mliver48, 2hr sampling rate mouse liver time course: mliver24, Zebrafish larval and liver time courses: zlarva and zliver. (B) Phase of rhythmic bZIP transcripts expressed in same datasets as Fig 5A. (C) Phase of rhythmic bHLH transcripts expressed in same datasets as Fig 5A. (D) *hand2* transcript profile in mouse liver and Zebrafish liver and larva. (E) *mitfa* transcript profile in mouse liver and Zebrafish liver and larva. (F) *usf1* transcript profile in Zebrafish liver and larva.

Although the majority of the rhythmic genes detected in the mouse liver and the Zebrafish liver and larval datasets are mostly different (Figs 2A, 3A and 4E), these datasets share clusters of co-regulated genes (Figs 2D and 3C) and some general features related to the level of common/tissue specific gene expression (Fig 4B and 4D). In the limited comparative analysis performed thus far, the emerging picture is that datasets share clusters of co-regulated genes sometimes expressed in the same phase range or at completely different time of the day. An additional observation is that although we identified several hundreds of co-regulated genes, there are far more genes that were only rhythmic in only a single time course. Are these genes uniquely rhythmic in Zebrafish liver or their respective tissue or just not rhythmic in our limited survey? It is possible that each dataset represents a specific ensemble of clock outputs even if they originated from the same tissue as others. Such possibility would necessitate the core clock network to be very plastic but at the same time still be controlled by the same core mechanism. This scenario would involve a conserved core clock mechanism but the plasticity would implicate a diversity of downstream regulators. Several regulatory scenarios are possible. In one of those, the circadian core cis-elements could be controlled by the core circadian clock factors but each condition or tissue could also express competing regulators at these core cis-elements. This scenario would generate specific rhythmic array of outputs expressed at different times. We generated a list of all rhythmic regulators and graphed the phases of regulator families associated with the three central core clock cis-element (E box (bHLHs binding site), D box (bZIPs binding site) and RRE (nuclear receptors response element))(Fig 5A–5C) [56]. In the two mouse liver and the Zebrafish liver and larval datasets, TFs associated with the three central clock cis-elements were found to oscillate at almost all phases of the day (Fig 5A–5C). Although the identity of these TFs is not conserved between the datasets, all datasets rhythmically contain waves of potential E box interactors at all time of the day. From a core cis-element (DNA motif) point of view, this finding suggests that very diverse outputs could be generated by different TFs associated to the same TF families in coordination with the core clock TFs. To confirm that some non-core clock TFs are rhythmic in some tissues but not in others, we performed a Zebrafish larva time course for comparison with the Zebrafish liver dataset. 5 and 6 days old Zebrafish larval were sampled in biological duplicates at 2hrs intervals for 2 days and transcript were quantified using the Affymetrix QuantiGene platform (Fig 5D–5F and S2A–S2C Fig). We selected three examples from the bHLH family displaying rhythmic expression in different dataset but not others. The *hand2* transcript cycles in Zebrafish and mouse livers but not in Zebrafish larva (Fig 5D and S2A Fig for SD), the *mitfa* transcript cycles in Zebrafish larva and mouse liver but not in Zebrafish liver (Fig 5E and S2B Fig for SD and significance) and the *usf1* transcript cycles in both the Zebrafish larva and liver (Fig 5F and S2C Fig for SD and significance). If the expression pattern is a reflection of protein activity, these different bHLH factors could produce different rhythmic gene clusters by promoting or competing with the core clock factors in a condition dependent manner.

## Conclusion

Despite commonalities between datasets the overlap between the same tissues from the same species display a rather small overlap [40]. One constant finding is that the overlapping group includes core clock genes [14]. This feature is often used to leverage circadian transcriptomic studies toward the identification of novel clock genes [14]. The low overlap between output networks from different studies is not in line with the assumption that billions of years of evolution must have led to a robust recurring set of oscillating outputs.

Several reasons can explain this low overlap. First, all studies rely on algorithms to call rhythmic transcripts with their own inherent accuracy level. Identifying the perfect algorithm is an intense area of research and at the moment the community has not converged on a specific choice [34]. The use of different algorithms could explain a part of the low overlap. A second important factor is the sampling frequency. In order to accurately identify rhythmic genes, it was found that sampling frequency of 1 sample/hour is largely superior as compared to 1 sample per 2 or 4 hours [10]. While economic reasons often dictate the favored choice, the most common sampling frequency is 1 sample per 4 hours. This choice can lead to the identification of fewer cycling transcript with a higher ratio of false positives. Studies analyzing circadian datasets using the same algorithm also identified low overlap between dataset from different laboratories [40].

The review of circadian transcriptomics studies reveals two alternative possibilities for this observed low overlap. The first one is that a large proportion, possibly all, genes are rhythmic and inappropriate sampling rate and algorithm hampers us to fully grasp the exact output network. The second alternative is that each study provides an expression of the circadian clock output network channeled by the nature of the environment in which the specimen was maintained. Variations in soil, food type, lighting, temperature, social interaction, microbiome will be incorporated in the expression of the circadian output that will be an adaptive response to the environment [3]. In this case, the observed network is exclusive to this specific environment. Depending on the conditions, some cluster of genes may be incompatible with clusters from other studies (e.g. different diets). This second alternative would better explain why there is always a substantial number of diurnall/circadian-controlled transcription factors (CCTFs) being expressed rhythmically with no clear role in the core clock mechanisms. These CCTFs may participate in an adaptive gating system controlling the output network.

In order to explore the circadian output network, we use a comparative transcriptomic approach. To leverage circadian mouse liver datasets generated by the community, we selected the same organ in the vertebrate Zebrafish *Danio rerio*. Our study reveals that the Zebrafish liver rhythmic transcriptome is of similar size as its mammalian counterparts, with phases covering the whole spectrum with two major clusters. Comparative analyses between other Zebrafish time courses revealed tight regulation of the core clock genes and phase shift of other groups. Comparison with mouse time course also highlighted similarities between the core clock set and shifting group of output genes. Comparison with tissue datasets identified a liver identity and features associated with specific group of tissues. Comparison of TF families revealed that the lack of overlap between output genes list is nevertheless associated with a similar size array of TFs from the same family oscillating at similar times. These features help inform on the potential global nature of the vertebrate output network and confirm the potential of Zebrafish liver and larva stage as model to build upon the actual knowledge from rodent and human to better investigate the overall circadian output network.

## Supporting Information

S1 Fig Correlation analysis of phase comparison graphs. (A) Scatter plot of the recalculated phases between the common transcripts found by HAYSTACK and JTK_Cycle. For correlation analysis, phases were recalculated in order to take into account phases close to the dawn transition (eg. ZT23 and ZT0). For phase difference larger than 12hrs, 24hrs was added to the smaller of the two phases. This adjustment extended the phase scale outside 24hrs. (B) Phase change distribution (hrs) between HAYSTACK and JTK_Cycle. (C) Scatter plot of the recalculated phases between Zebrafish liver and larva. Phases were recalculated as described in (A). (D) Phase change distribution (hrs) between Zebrafish liver and larva. (E) Scatter plot of the recalculated phases between Zebrafish and mouse livers. Phases were recalculated as described in (A). (F) Phase change distribution (hrs) between Zebrafish and mouse livers.
(TIF)

S2 Fig **Relative transcript level of *hand2* (A), *mitfa* (B) and *usf1* (C) in Zebrafish larva.** Detailed analysis of transcript level using the QuantiGene platform. Circles indicate the mean of the relative expression over the *timeless* reference gene. The error bars represent the standard deviation between the biological duplicates. The Significance (p<0.05) from t-test between acrophases and troughs are indicated with a star (*usb* at ZT2-14 = 0.06 and ZT26-38 = 0.02 and for mitfa at ZT6-18 = 0.09 and ZT30-42 = 0.02).
(TIF)

S1 Table Rhythmic genes in Zebrafish liver (transcript, genes and mouse orthologs). (XLSX)

S2 Table Zebrafish and mouse transcription factor list. (XLSX)
