# Supplementary figures and images for: Comparative Analysis of Vertebrate Diurnal/Circadian Transcriptomes
Source: PLoS One. 2017 Jan 11;12(1):e0169923. doi: 10.1371/journal.pone.0169923 (PMC5226840; doi:10.1371/journal.pone.0169923)

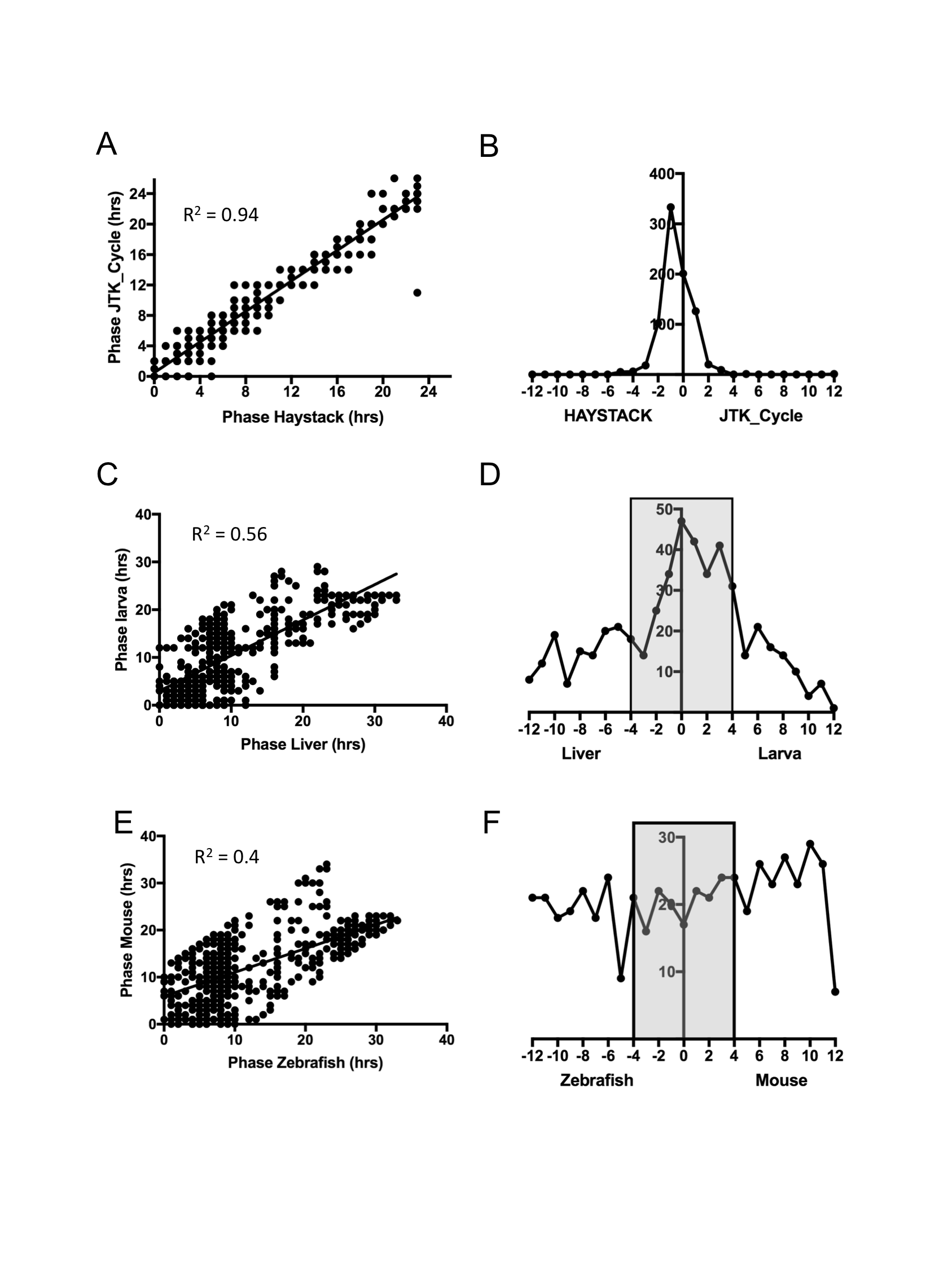

Supplement: S1 Fig — (A) Scatter plot of the recalculated phases between the common transcripts found by HAYSTACK and JTK_Cycle. For correlation analysis, phases were recalculated in order to take into account phases close to the dawn transition (eg. ZT23 and ZT0). For phase difference larger than 12hrs, 24hrs was added to the smaller of the two phases. This adjustment extended the phase scale outside 24hrs. (B) Phase change distribution (hrs) between HAYSTACK and JTK_Cycle. (C) Scatter plot of the recalculated phases between Zebrafish liver and larva. Phases were recalculated as described in (A). (D) Phase change distribution (hrs) between Zebrafish liver and larva. (E) Scatter plot of the recalculated phases between Zebrafish and mouse livers. Phases were recalculated as described in (A). (F) Phase change distribution (hrs) between Zebrafish and mouse livers. (TIF) [file pone.0169923.s001.tif]

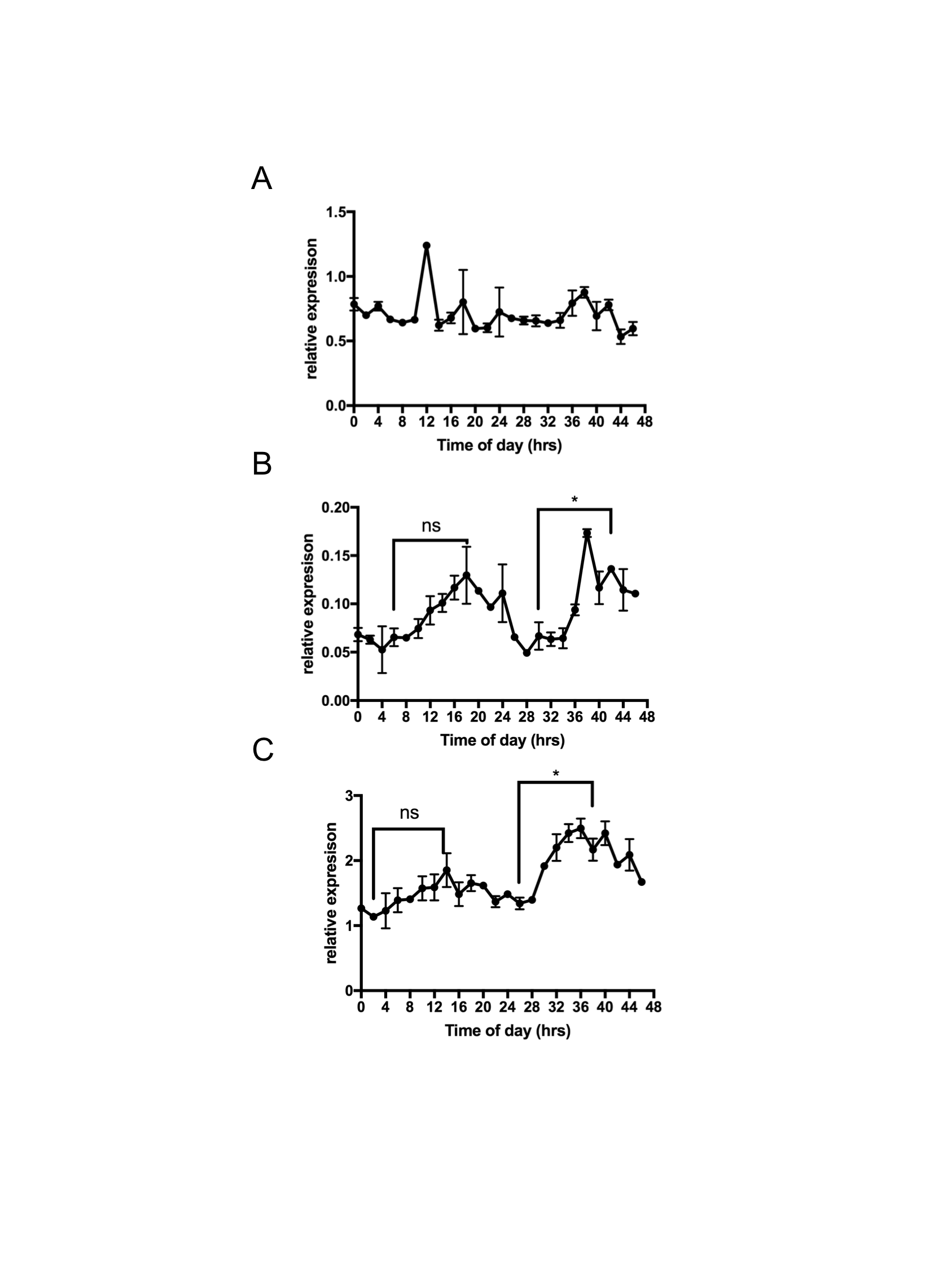

Supplement: S2 Fig — Relative transcript level of hand2 (A), mitfa (B) and usf1 (C) in Zebrafish larva. Detailed analysis of transcript level using the QuantiGene platform. Circles indicate the mean of the relative expression over the timeless reference gene. The error bars represent the standard deviation between the biological duplicates. The Significance (p<0.05) from t-test between acrophases and troughs are indicated with a star (usb at ZT2-14 = 0.06 and ZT26-38 = 0.02 and for mitfa at ZT6-18 = 0.09 and ZT30-42 = 0.02). (TIF) [file pone.0169923.s002.tif]
